# Diet and Life Stage-Associated Lipidome Remodeling in Atlantic Salmon

**DOI:** 10.1021/acs.jafc.0c07281

**Published:** 2021-03-23

**Authors:** Yang Jin, Thomas Nelson Harvey, Zdenka Bartosova, Sahar Hassani, Per Bruheim, Simen Rød Sandve, Jon Olav Vik

**Affiliations:** † Center of Integrative Genetics (CIGENE), Norwegian University of Life Sciences, 1430 Aas, Norway; ‡ Department of Biotechnology and Food Science, Norwegian University of Science and Technology, 7491 Trondheim, Norway; § Faculty of Chemistry, Biotechnology and Food Science, Norwegian University of Life Sciences, 1430 Aas, Norway

**Keywords:** lipidomics, atlantic salmon, life stage, long-chain highly unsaturated fatty acids, vegetable oil

## Abstract

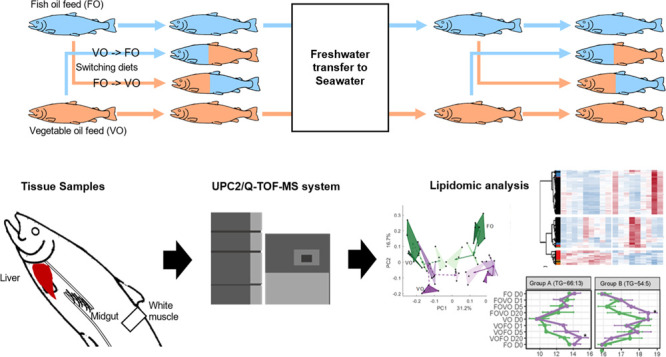

Salmon is an important source of long-chain highly unsaturated fatty acids (LC-HUFAs) such as 22:6*n*-3 [docosahexaenoic acid (DHA)]. In the present study, we conducted two identical experiments on salmon in freshwater (FW) and seawater (SW) stages, with a diet switch from fish oil (high in LC-HUFA) to vegetable oil (low in LC-HUFA) and vice versa. Our aim was to investigate the diet and life stage-specific features of lipid uptake (gut), processing (liver), and deposition (muscle). The lipid composition changed much faster in the gut of SW fish relative to FW fish, suggesting that the former had a higher rate of lipid absorption and transport. SW fish also had higher expression of phospholipid synthesis and lipoprotein formation genes in the gut, whereas FW fish had higher expression of lipid synthesis genes in the liver. All phospholipids except PC-44:12 and PE-44:12 were less abundant in SW, suggesting that SW fish have a higher requirement for DHA.

## Introduction

Farmed Atlantic salmon (*Salmo salar*) is a popular fish species for human consumption since it contains high amounts of long-chain highly unsaturated fatty acids (LC-HUFAs) such as docosahexaenoic acid (22:6*n*-3, DHA) and eicosapentaenoic acid (20:5*n*-3, EPA). LC-HUFAs are also essential for the fish because they are key components for membrane fluidity, eicosanoid production, and neural tissues.^1−3^ The levels of LC-HUFA in salmon depend not only on the dietary supply but also on the biosynthetic capacity of the fish, which can vary between individuals and life stages.^4,5^ The absorbed and biosynthesized LC-HUFAs could be incorporated in the forms of different lipid species and have different biological properties;^6^ however, this has not been extensively studied in salmon.

Atlantic salmon is anadromous, implying that it migrates from freshwater (FW) to seawater (SW) and back throughout its life cycle. During this migration, the fish undergo large-scale morphological, physiological, and endocrinological changes to adapt to differences between FW and SW habitats. A successful migration between FW and SW involves the coordination of several hormones such as growth hormones,^7^ thyrotropin,^8^ cortisol, and prolactin.^9^ These hormones not only help the fish to tolerate salinity changes between SW and FW habitats but also alter their metabolism of proteins, lipids, and carbohydrates, likely as an adaptation to the different dietary profile of marine prey.^10^

Salmon live in high latitudes where SW ecosystems often have higher productivity than FW.^11^ The richer food availability at the sea could lower the salmon’s requirement for *de novo* lipogenesis but increase lipolytic activity in the body.^12^ A recent study on comparative transcriptomics has also suggested that SW salmon had increased gene expression in pathways for lipid absorption but decreased gene expression in pathways for lipid synthesis as compared to FW salmon.^5^ Differences in food availability and the lipid metabolism between FW and SW could lead to different requirements for essential lipids and LC-HUFAs. Fish oil (FO) high in LC-HUFAs is traditionally used in salmon aquaculture; however, recently, cheaper and more sustainable vegetable oil (VO), naturally devoid of LC-HUFA, has become a popular alternative. This dietary deficiency is known to cause up-regulation of LC-HUFA synthesis in salmon, but due to differences in absorption and biosynthetic capacity, SW and FW salmon could have different tolerance to VO diets.

The use of genomics and transcriptomics has been increasingly adopted across multiple aquaculture species including salmon.^13−15^ These omics techniques have greatly improved the accuracy of selection in fish breeding^16^ and the understanding of the standard metabolism in fish.^17^ Lipidomics aims to take a snapshot of all lipid species present in a sample at a given time. This is a powerful tool for assessing global changes in the lipidome and has been successfully applied to study immunology in grouper^19^ and toxicology in salmon^20^ and to assess the freshness of commercial salmon filets.^18^ Lipidomics has not yet been widely utilized in nutritional studies of aquaculture species, with most studies employing traditional lipid profiling approaches such as fatty acid methyl ester analysis^21^ and thin-layer chromatography.^22^ Lipidomics offers a distinct advantage over these techniques because it measures the lipid class, species, and abundance in a single direct measurement,^23^ offering a broader population of structural information that is important for nutrition. For example, a study in cod suggests that the acylation of LC-HUFA onto different lipid classes, rather than LC-HUFA itself, is more important for the growth and survival of marine larvae.^24^

In the present study, we performed a diet switch experiment where salmon was fed either an FO diet or a VO diet from initial feeding and then was switched to the opposing diet in both FW and SW life stages. Our aim was to investigate the kinetics of lipidome changes in SW and FW salmon after switching diets. Based on differences in dietary availability in FW and SW environments, we hypothesized that the SW salmon have a higher capacity for lipid absorption, resulting in a more rapid lipidomic response to changes in feed in SW environments. Additionally, LC-HUFAs could be incorporated into different lipid species in SW salmon as compared to FW salmon. By comparing lipid species which contained LC-HUFAs between SW and FW fish, we were able to assess differences in LC-HUFA requirements between the two stages of salmon.

## Materials and Methods

### Feeding Trial

Atlantic salmon was either fed an FO diet containing ∼17% DHA and EPA of the total lipid fraction or a VO diet containing ∼3.8% DHA and EPA from initial feeding (Table S1).^5^ Except for lipid sources, other ingredients were identical between the FO and VO diets (Table S1).^5^ Total lipids were kept constant between the FO and VO diets; however, a higher lipid proportion (31%) was used in SW feed as compared to FW feed (22%). This is standard practice in the aquaculture industry to use increasing lipid as the fish grows in order to maintain optimal growing conditions by decreasing the digestible protein to digestible energy ratio.^25^ When the fish reached ∼50 g in FW, a group of fish was transferred for a diet-switch experiment, where the fish was switched to the contrasting diet (VO to FO or vice versa). Gut, liver, and muscle samples were taken from fish at days 0 (before diet switch), 1, 5, and 20 after diet switch. The remaining fish began the smoltification process 2 weeks after the FW sampling.^5^ After 10 weeks of smoltification, the fish was transferred to SW and cultivated for another 3 weeks. The diet-switch experiment was then repeated in SW salmon (∼200 g). The fish were starved on the mornings of each sampling day. Individual fish were euthanized by a blow to the head, and then, samples of the midgut, liver, and muscle were flash-frozen in liquid nitrogen. All tissue samples were stored at −80 °C before further lipidomic and RNA-seq analyses.

### Lipid Extraction and Lipidomic Analysis

Four individuals per group (*n* = 4, 2 fish per tank × 2 replicate tanks) were used for lipidomic analysis. A two-step extraction and a solvent system based on the Folch method were applied to isolate lipids from the salmon gut, liver, and muscle tissue.^26^ A Precellys24 bead homogenizer equipped with a Cryolys temperature controller (Bertin Technologies AS, Montigny-le-Bretonneux, France) was employed to disrupt and homogenize the tissue samples. The gut (20 mg), liver (50 mg), and muscle (50 mg) tissue were homogenized with zirconium oxide beads (0.5 ± 0.01 g, 1.4 mm) in 500 μL of a cold mixture of chloroform/methanol (2:1, v/v). The samples were kept frozen during cutting and weighing. Three cycles of bead beating at 6500 rpm for 30 s with an intermediate 15 s pause were needed to obtain a homogeneous sample. After that, another portion of 500 μL of a cold mixture of chloroform/methanol (2:1, v/v) was added to the sample, and the tubes were shaken for 10 min at 1000 rpm using a thermoshaker (Thermal shake lite, VWR International AS, Bergen, Norway). Phase separation was induced by adding 200 μL of water. The shaking step was repeated (10 min, 1000 rpm), and phase separation was achieved by centrifugation for 5 min at the maximum speed (13400 rpm) using a small centrifuge (MiniSpin, Eppendorf). A lower layer (400 μL) containing lipids was collected, and the sample was re-extracted with the addition of 400 μL of a cold mixture of chloroform/methanol/water (86:14:1, v/v); the shaking and centrifuge steps were repeated. Subsequently, 650 μL of the lower layer was collected and pooled with the first lipid extract. The final extract was cleared of cell debris with a syringe filter with a GHP membrane (0.2 μm, 13 mm, Acrodisc, Pall Laboratory, USA) and stored at −80 °C prior to further lipidomic analysis. Dichloromethane was used as a sample diluent prior to injection.

A nontarget analysis of lipids was performed using an ultraperformance supercritical fluid chromatographic system UPC^2^ coupled to a quadrupole time-of-flight mass spectrometer SYNAPT G2-S HDMS (UPC^2^-MS/MS, Waters AS, Milford, MA, USA). A chromatographic method previously described by Lísa and Holčapek^27^ was adopted and modified.^54^ In general, the separation was carried out on an Acquity UPC^2^ BEH column (100 mm × 3 mm, 1.7 μm) protected with a BEH VanGuard precolumn (2.1 × 5 mm). The UPC^2^ separation system was equipped with a binary pump, a convergence manager, a column heater, an autosampler, and an auxiliary pump. The chromatographic system was connected to the mass spectrometer via a flow splitter kit consisting of two T-pieces enabling control of the back pressure and infusion of a makeup liquid. Pressurized CO_2_ was used as mobile phase A, and methanol/water (99:1, v/v) containing 30 mM ammonium acetate was used as mobile phase B. The gradient of mobile phase B followed the scheme 0 min, 1%; 4.0 min, 30%; 4.4 min, 50%; 6.25 min 50%; 7.25 min, 50%; 7.35 min, 1%; 8.50 min, 1%. The makeup liquid consisted of methanol/isopropanol/water (50:49:1, v/v/v), and its flow rate was set to 0.2 mL/min. The column was maintained at 50 °C, the flow rate of the mobile phase was set to 1.9 mL/min, and the automated back-pressure regulator was set to 1800 psi.

The mass spectrometer was equipped with an ESI source operated in the positive mode allowing ionization of CE, COH, TG, DG, MG, CER, GluCer, GalCer, PG, PE, LPE, PC, LPC, and SM. A data-independent acquisition technique MS^E^ was applied for data acquisition, and the collision energy ramped from 20 to 30 eV. The MS tuning parameters were set as follows: capillary voltages of 3.0 kV, the source temperature of 150 °C, the sampling cone of 40 V, the source offset of 60 V, the cone gas flow of 50 L/h, the desolvation temperature of 500 °C, the desolvation gas flow of 850 L/h, and the nebulizer gas pressure of 4 bar. Data were obtained over the mass range of 50–1200 Da, and the resolution of the mass spectrometer was 20,000.

Data were obtained using a MassLynx 4.1 software program (Waters Corporation). Raw data were processed employing Progenesis QI software (Nonlinear Dynamics, Waters) equipped with a Lipid Maps Structure Database^28^ and a LipidBlast database^29^ for lipid identification. In supercritical fluid chromatography, the lipids are separated according to their headgroup and each class is eluted in a narrow discrete zone. Lipid classes were identified using MS and retention characteristics. Filters specifying the retention window and *m*/*z* ranges for each individual class have been applied to designate compounds belonging to each lipid class.^54^ Retention times of lipid classes were then confirmed by comparison with one or two standards per class. The identification of individual lipid compounds is based on the following characteristics: retention time of the appropriate lipid class, accurate mass (ppm error <5), isotope pattern similarity (>80%), and the fragmentation pattern. The lipid nomenclature and shorthand notation described by Lipid Maps^30,31^ and Liebisch et al.^32^ were used in this paper. The raw data were normalized using the default method in the Progenesis QI software, referred to as Normalize to All Compounds (https://www.nonlinear.com/progenesis/qi/v3.0/faq/how-normalisation-works.aspx).

Further analysis of the normalized abundance was performed using R (version 3.4.1).^33^ Two-way ANOVA with Tukey’s HSD was used to test the effect of diet (D0 FO, D1 FOVO, D5/D6 FOVO, D20 FOVO, D0 VO, D1 VOFO, D5/D6 VOFO, and D20 VOFO) and life stages (SW and FW) on content of each lipid species. Samples of different tissues were analyzed separately. Differences were considered significant at |Log 2 FC|>1 and *p* < 0.05. All figures were made using R package ggplot2.^55^

### Multiblock Data Analysis on Whole Lipidomes of Fish

Multiblock methods are data modeling approaches that can maintain block-structured data sets, such as blocks of lipidomic data from different lipid classes.^34^ This was done in order to enable running a simultaneous analysis of the data belonging to the different lipid classes with the aim of detecting between and within class variations of lipids. In the present study, the lipidomic data were first aligned in such a way that samples (i.e., fish) were represented by rows and variables (i.e., lipid species) by columns. The data were then grouped into the data blocks of triacylglycerols (TGs), diacylglycerols (DGs), phosphatidylethanolamines (PEs), and phosphatidylcholines (PCs) and a data block of lipid species belonging to other lipid classes (e.g., sphingolipids and monoacylglycerols), resulting in a total of five data blocks. The data blocks were mean-centered by subtracting the mean of each lipid class and were then scaled by dividing each lipid signal by its standard deviation. The different data blocks in the multiblock data set were then block-wise scaled (with respect to the sum of squares) prior to data modeling. This procedure results in an equal total sum of squares for the different data blocks with different numbers of variables and gives every block the same importance during the data modeling process.

The sample variation patterns are visualized by running consensus principal component analysis (CPCA) on the multiblock data set which results in two types of score plots: the global score plot and block score plots. The global score plot represents an overview of the sample variation pattern shared among the blocks of data (hereby the pattern that most of the lipid classes share). The block score plots illustrate the contribution of every data block to the detected shared pattern. In this study, the block score plots represent how much of the shared pattern among the lipid classes is represented within every lipid class. The variable variation patterns are visualized in the correlation loading plots where the lipid–lipid interactions can be detected. Correlation coefficients between the original lipid measurements and the principal components are unit-free parameters and indicate dependencies between the principal components and the lipid measurements. The sum of the squares of the correlation coefficients gives a relative amount of explained variance and is visualized by an outer and inner circle with radii of 1 and 0.5 representing 100 and 50% explained variances, respectively.

### RNA-seq Analysis

A previous study has compared the transcriptome between FW and SW salmon by using fish from the same experiment.^5^ In the present study, we took their raw count tables and performed differential expression analysis using the R package edgeR.^35^ Eight individuals per group (4 fish per tank × 2 replicate tanks) were used for RNA-seq analysis. Differential expression analysis was applied between every two conditions and yielded log 2-fold change (Log 2 FC) and a false discovery rate (FDR, adjusted *p*-value) for each gene. Genes with FDR <0.05 were considered differentially expressed genes. Normalized gene counts in the form of transcript per million (TPM) values were used for visualizing expression levels between the diet and life stages.

## Results

### Lipid Class Dynamics between Tissues and Life Stages

**Figure 1:**
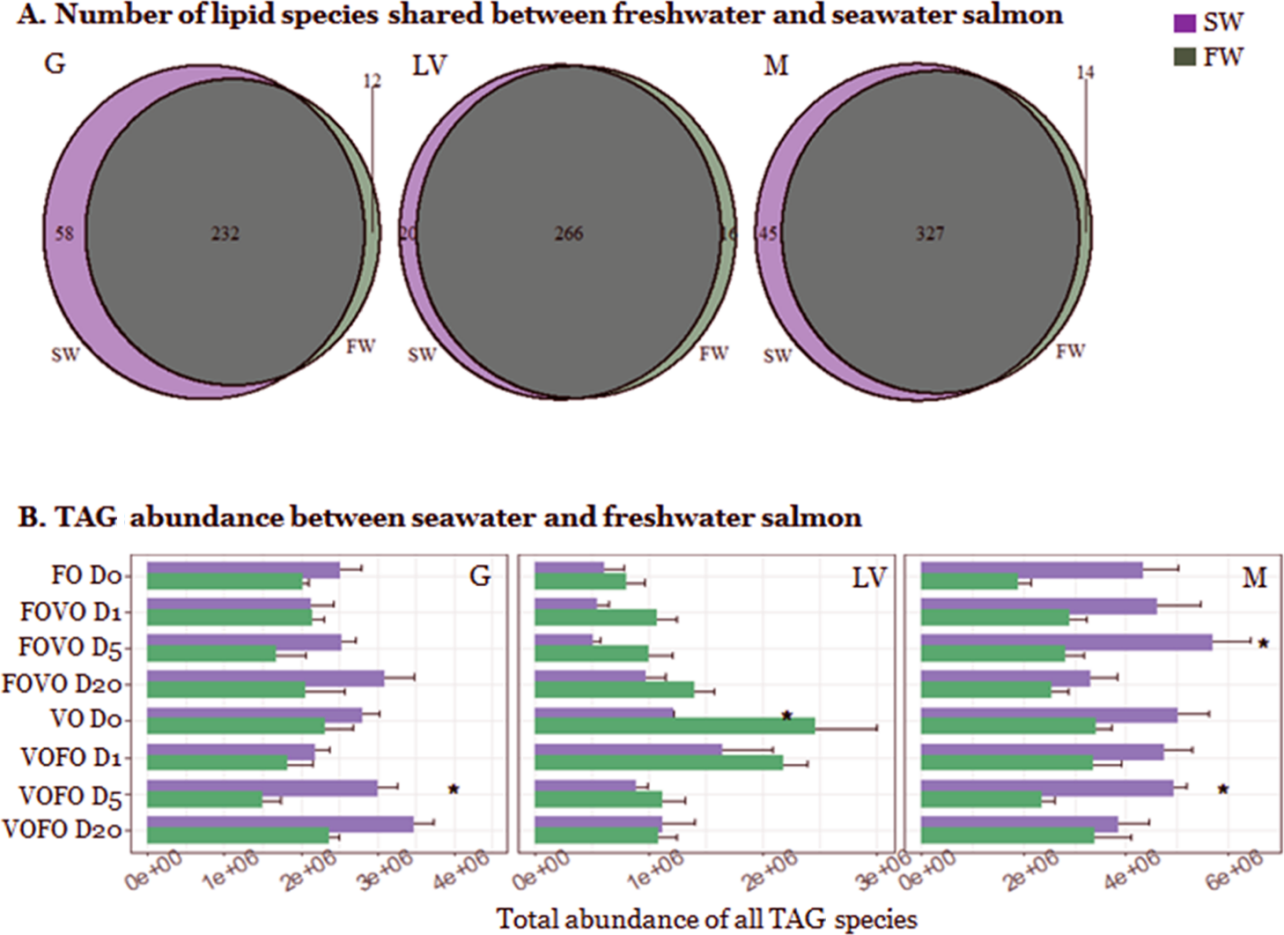
Overview of lipid species between SW and FW salmon. (A) Total number of lipid species between SW and FW in each tissue. (B) Total abundance of all TG species between SW and FW salmon before diet switch between FO and VO (FO D0 and VO D0) and at 1, 5, and 20 days after diet switch from FO to VO (FOVO) and vice versa (VOFO). Asterisks indicate significantly (*p* < 0.05) different abundance of TG between FW and SW under the same dietary treatment (*n* = 4 per tissue per group).

An average number of 300 lipid species were detected in each gut, liver, and muscle tissue, with which over 90% of the lipids were shared between FW and SW salmon (Figure 1A). Compared to other lipid classes, triacylglycerols (TGs), phosphatidylcholines (PCs), and phosphatidylethanolamines (PEs) covered more than 90% of the total abundance of lipid (Figure S1). The total abundance of PCs and PEs was similar between SW and FW fish for all three tissues, while the content of TGs was different. In general, salmon in SW had higher abundance of TGs in the gut and muscle, while fish in FW contained more TGs in the liver (Figure 1B). Switching to the VO diet increased the total amount of TGs in the liver of both SW and FW fish (Figure 1B). However, the increase of TGs was much larger in FW salmon; therefore, it had a significantly (*p* = 0.04, Log 2 FC = 1.02) higher amount of TGs than SW fish when constantly fed a VO diet (day 0 VO).

### Lipid Remodeling Following Diet Switch

**Figure 2:**
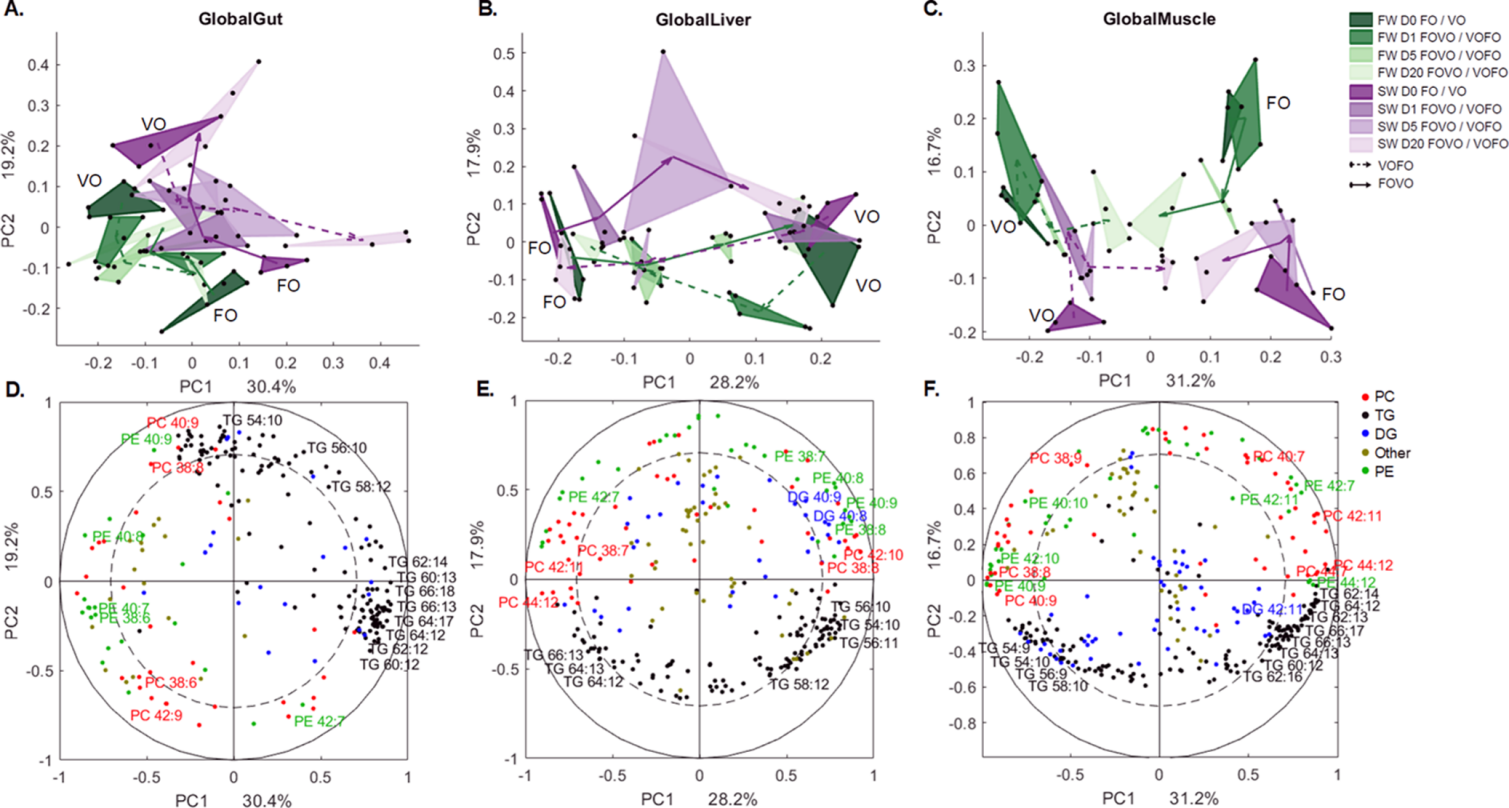
Overview of lipid species between SW and FW salmon. The top three plots are CPCA global score plots for common lipid species between SW and FW salmon during diet switch. CPCA score plots were generated separately for the liver, gut, and muscle tissues. The bottom plots are correlation loading plots corresponding to each CPCA global score plot. Only lipid species with higher levels of unsaturation are labeled. All lipid species were grouped into five lipid classes, PC, TG, DG, PE, and other lipid classes (Other). The same diet labeling was used as in Figure 1 (*n* = 4 per tissue per group).

The global score plot of the multiblock analysis showed a clear separation of all lipid profiles between salmon fed VO and FO for all three tissues (Figure 2). The composition of lipid species changed more rapidly in the gut and liver than in the muscle. 20 days after switching diets, the lipid profiles in the liver and gut of the FOVO or VOFO fish had reached a “steady-state” lipid level similar to that of control fish that were not diet-switched (Figure 2A,B); however, this was not the case for the muscle (Figure 2C). We did not observe a clear difference in the speed of lipid remodeling between FOVO and VOFO. The separation of lipids between SW and FW fish was generally more clear in the gut and muscle than in the liver (Figure 2A–C). The block score plot of each lipid class showed that PCs and PEs had better separation for SW and FW fish compared to neutral lipid classes (Figure S2).

The correlation loading plot (Figure 2D–F) shows clear separation of lipid species by both diet (PC1) and water (PC2). TGs were generally highly represented in the gut and muscle of SW salmon while poorly represented in the liver of SW salmon. On the other hand, most of the PLs were positively correlated to the liver but negatively correlated to the gut and muscle of SW fish. We have also observed representative lipid species for different dietary treatments. The TGs with a higher chain length (*C* ≥ 60) and more double bonds (*n* = 12–18) were more associated with fish under FO diet. Fish fed a VO diet also contained certain amounts of polyunsaturated TGs but with a relatively shorter chain length (*C* < 60) and lower levels of unsaturation (*n* = 10–12), such as 56:10, 54:10, and 58:10. Fish fed FO or VO diets also had different compositions of phospholipid species. For example, PC-42:11 and PC-44:12 were higher in fish fed a FO diet, while PC-40:9 and PC-42:10 were higher in VO-fed fish.

Our lipidomic approach unfortunately cannot identify the composition and stereospecific position of fatty acids on each lipid species. For this reason, we analyzed the total fatty acid composition for the same fish used for lipidomic approaches (Figure S3). This could help us estimate the fatty acid composition of each lipid species. For example, PC-44:12 was likely composed of two 22:6*n*-3, which was more abundant in fish fed an FO diet. Fish fed a VO diet contained a combination of 22:6*n*-3 and 18:3*n*-3; therefore, they have higher levels of PE-40:9 and PC-40:9.

### Lipid Remodeling Across Life Stages

**Figure 3:**
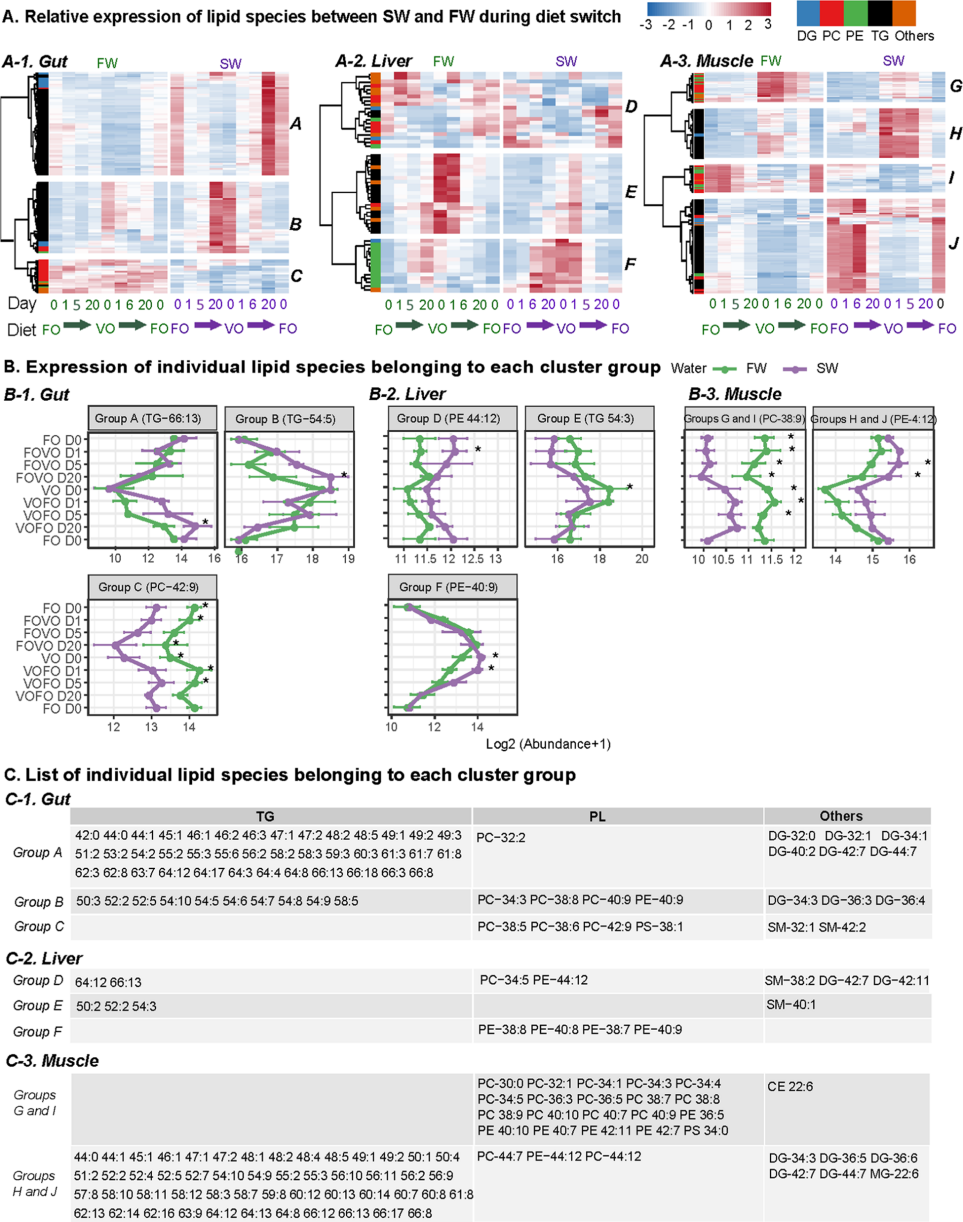
Comparison of individual lipid species between SW and FW during diet switch. (A) Heatmap of lipid species which were significantly (*p* < 0.05) different between SW and FW salmon at any sampling point. Abundance of each lipid species was scaled among all sampling points and life stages (row-scaled). Each heatmap was split into different groups based on cluster similarities. Samples were split into FW (green) and SW (purple) groups. For each group, columns from left to right indicate samples of day 0 fish oil (FO), day 1 switching from fish oil to vegetable oil (FOVO), day 5 (day 6 for SW) FOVO, day 20 FOVO, day 0 vegetable oil (VO), day 1 switching from vegetable oil to fish oil (VOFO), day 5(6) VOFO, day 20 VOFO, and D0 FO. All lipid species were grouped into five lipid classes, PC, TG, DG, PE, and other lipid classes (Other). (B) Plot of individual lipid species representative of lipid changes in each cluster group. Asterisks indicate significantly (*p* < 0.05) different lipids between SW and FW salmon from each dietary group. (C) Other identified lipid species which were clustered in each group (*n* = 4 per tissue per group).

In order to understand life-stage-associated changes of lipids in salmon, we compared the content of individual lipid species between SW and FW over 20 days of diet switch. Out of ∼300 lipid species, 125 species in the gut were significantly (|Log 2 FC|>1 and *p* < 0.05) different between SW and FW in our experiment (Figure 3A-1). In general, the gut tissue had the fastest lipid composition remodeling following diet switch. Furthermore, this remodeling was faster in the gut of SW fish compared to FW fish. This pattern was mostly driven by the more rapid change of TGs in SW (Figure 3A-1,B-1, groups A and B). Among the fast-changing lipids in SW, we also found a few PLs such as PC–38:8, PC–40:9,and PE–40:9 (group B in Figure 3). However, for most PLs, such as PC-42:9, the general trend was a much higher baseline level in FW regardless of diet switch (group C in Figure 3).

Lipids in the liver were more consistent across life stages, with only 55 lipid species significantly different between the livers of FW and SW salmon. Other than this, 17 significant TGs were more abundant in FW when under VO diet, while 12 PE species including PE-38:8 and PE-40:9 were more abundant in SW fish under the VO diet but not the FO diet (Figure 3A-2–C-2). Interestingly, the level of PE-44:12 (DHA-DHA-PE) was generally more abundant in SW than in FW (Figure 3B-2, group D).

Compared to the liver and gut, the muscle had the highest number (191) of differentially abundant (|Log 2 FC|>1 and *p* < 0.05) lipid species between SW and FW salmon. Most of the PLs were more abundant in the muscle of FW salmon compared to SW salmon with the exception of PC-44:12 and PE-44:12, which were more abundant in SW (Figure 3A-3–C-3). All TG species were more abundant in the muscle of SW fish compared to FW fish. Unlike in the liver and gut, in the muscle, differences in lipid composition between FW and SW were not largely affected by diet switch.

Fatty acid analyses revealed that 18:3*n*-3 and 18:2*n*-6 increased, while LC-HUFA decreased in the gut, liver, and muscle of the fish after switching to a VO diet. However, no differences in fatty acid composition were observed between SW and FW fish fed the same diet (Figure S3).

### Expression of Key Genes Involved in the Lipid Metabolism

**Figure 4:**
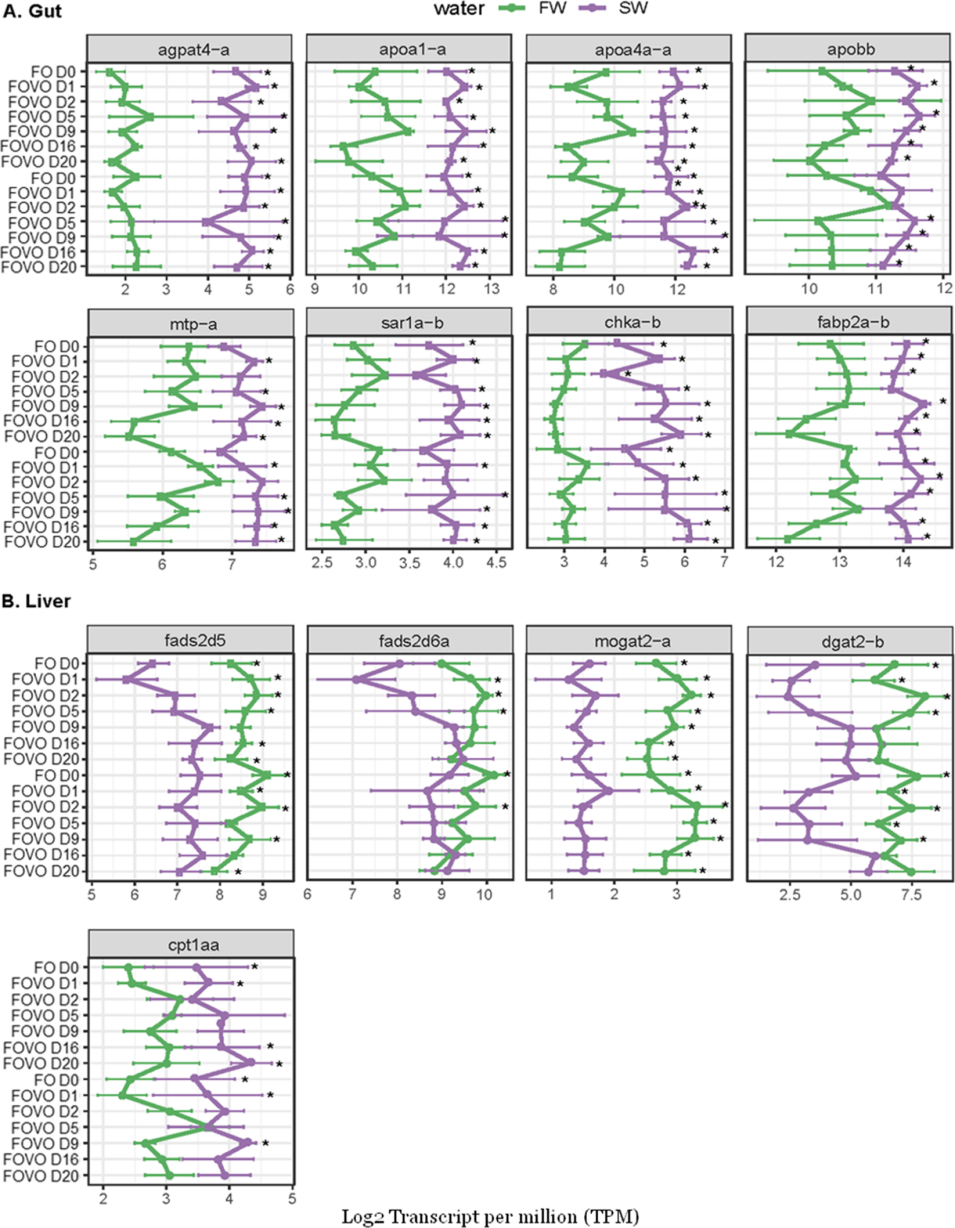
Expression of key genes involved in lipid synthesis and transport between SW and FW salmon. Gene expression was compared in the form of log 2 TPM between SW and FW fish fed FO and VO diets before diet switch and 1, 2, 5, 9, 16, and 20 days after diet switch from FO to VO (FOVO) and vice versa (VOFO). Asterisks indicate significantly (*p* < 0.05) different abundance of TG between FW and SW in each diet group (*n* = 8 per tissue per group).

In parallel to the increased rate of lipid remodeling in the gut of SW salmon, we observed higher steady-state expression levels of genes involved in lipid transport pathways (Figure 4A). These genes include *agpat*4*-a* and *chka-b*, involved in *de novo* synthesis of glycerophospholipid,^36,37^ genes *mtp-a*, *sar*1*a-b*, *apobb*, *apoa*4*a-a*, and *apoa*1*-a* involved in lipoprotein formation,^36^ and gene *fabp*2*a-b* involved in intracellular transport of fatty acids.^38^ In the liver, no difference was observed in the expression of genes involved in phospholipid and lipoprotein formation between the livers of SW salmon and FW salmon. However, the expression of genes *mogat*2*-a* and *dgat*2*-b* involved in TG synthesis and *fads*2*d*5 and *fads*2*d*6*a* involved in LC-HUFA synthesis had higher expression in FW than SW salmon. The expression of the key gene *cpt*1*aa* involved in fatty acid degradation was more highly expressed in SW salmon.

## Discussion

The present study has provided a systemic overview for the abundance and distribution of hundreds of lipids in Atlantic salmon and the dynamic remodeling of these lipids between diets and across life stages. Dietary availability (VO vs FO) determined to a large extent which lipid species salmon accumulated and synthesized. Dietary TGs in salmon are mostly digested into *sn*-2-monoacylglycerol (2-MAG) and free fatty acids (FFAs), which are then absorbed into enterocytes and re-synthesized into TG and PL before entering other parts of the body.^39,40^ The type of FFA which was esterified onto new TGs largely depends on its abundance in the cell. Compared to FO, VO is naturally devoid of DHA and EPA but contains higher amounts of α-linolenic acid (ALA, 18:3*n*-3) and linoleic acid (LA, 18:2*n*-6).^41^ This explains the higher amount of TG-66:18 (DHA–DHA–DHA) and TG-64:17 (DHA–DHA–EPA) in fish fed the FO diet, while fish fed the VO diet contained higher amounts of TG-54:9 (likely 18:3*n*-3-18:3*n*-3-18:3*n*-3) and TG-54:8 (likely 18:3*n*-3-18:3*n*-3-18:2*n*-6). Dietary PLs in fish are predominately digested by phospholipase A2 into 1-acyl-*sn*-phospholipid (lyso-PL) and FFA, which are then absorbed and re-synthesized into new PLs in enterocytes by lysophospholipid acyltransferase (LPAT).^3,36^ Our study has found higher amounts of PC-44:12 (DHA-DHA-PC) and PE-44:12 (DHA-DHA-PE) in salmon fed a FO diet, while fish fed a VO diet had higher amounts of PC-40:9 (likely 18:3*n*-3-DHA-PC) and PC-38:8 (likely 16:0-DHA-PC). The high PL-DHA in both VO- and FO-fed salmon suggests that LPAT preferentially esterifies LC-HUFA over other fatty acids. We also found that PL-44:12 and PC-44:12 increase in the muscle of SW fish regardless of the diet. Since the PL source (fish meal) in FO and VO diets was identical and since DHA is predominantly located in the *sn*-2 position of PLs,^42^ the different fatty acid composition between tissue PLs of SW and FW salmon suggests high levels of fatty acid remodeling on the *sn*-1 position of PL.

The most striking result from this experiment was the increased rate of lipid uptake in the gut of SW salmon. Salmon in SW drink water continuously to regain passive water loss due to high salinity.^43^ The absorption of water is mainly controlled by basolateral Na^+^/K^+^-ATPase, which also play an important role on alkalization of the luminal contents.^44^ The migration to SW changes the pH,^44^ osmolality, passage rate,^45^ and microbial composition^46^ in the intestinal tract, which could also have a large impact on lipid absorption. The increased rate of lipidomic remodeling in the gut also correlates to higher expression of genes involved in lipoprotein formation and transport pathways in SW salmon,^5^ suggesting that the fish also have a greater ability to transport lipids from enterocytes to the rest of the body.^36^ However, we observed neither faster incorporation nor higher gene expression in the liver and muscle of SW salmon. This implies that faster lipid absorption from the gut has little contribution to lipid distribution throughout the body and suggests a limited capacity of lipid uptake in the peripheral tissue.

Previous studies have suggested that lipogenesis in salmon decreases after migrating to SW.^5,12^ Our results support this interpretation because we find that FW salmon have higher overall levels of TGs than SW salmon and higher expression of genes in TG synthesis (*mogat* and *dgat*) and LC-HUFA synthesis (*fads*2*d*5 and *fads*2*d*6*-a*). The lower requirement of endogenous lipid synthesis in SW is probably due to higher capacity of lipid absorption and transport from the gut. Similar to previously published studies,^47,48^ we found that feeding a VO diet induces expression of genes in endogenous synthesis of fatty acid (*fasn* and *acc*) and cholesterol (*hmgcr*, *fdft*, *sqlea* etc.). This can explain the higher amount of liver TGs in VO-fed FW salmon compared to VO-fed SW salmon. The observed differences in the lipid metabolism between FW and SW salmon could reflect adaptive remodeling of the metabolism and physiology when the salmon migrates from FW to SW. Since ocean environments at higher latitudes are more productive than river environments,^11^ lipid requirements for growth and development of sea dwelling salmon are more readily met by the diet and fewer resources need to be allocated to endogenous lipid synthesis. In short, the switch from lipid-poor rivers to lipid-rich oceans corresponds to a switch from high-energy lipid synthesis in the liver to low-energy lipid absorption in the gut. Although the total lipid content was different between SW and FW diets, previous studies have found that increasing dietary lipid content has no effect on the expression of apolipoprotein A-I (*apoa*1),^56^ apolipoprotein A-IV (*apoa*4),^57^ microsomal TAG transfer protein (*mtp*),^58^ scavenger receptor class BI (*sar*1*-b*),^58^ and fatty acid binding protein 2 (*fabp*2)^57^ in fish. Since the genes were differentially expressed between SW and FW in our study, the expression differences on lipid transport and synthesis genes were more likely to be due to metabolic differences between SW and FW fish than differences in total lipid content between feeds.

The consistently lower level of most muscle PLs in large SW salmon (∼200 g) compared to small FW salmon (∼50 g) was likely an effect of increasing TGs since each lipid species was normalized by the total lipid amount in each sample. While incorporation of dietary fatty acid in TG generally follows a dilution model, incorporation in PL is much more complex.^49^ The levels of PC-44:12 (DHA–DHA–PC) and PE-44:12 (DHA–DHA–PE) were both more abundant in SW fish than in FW fish, suggesting a higher requirement for DHA in PL in SW. One common explanation for the high DHA in SW fish is that high PL-DHA can largely increase membrane fluidity, especially at lower temperatures.^50^ However, the water temperature was kept constant (∼8 °C) for both FW and SW during the diet-switch experiment. Thus, the higher accumulation of PL-DHA in the muscle of SW salmon could rather be associated with altered Na^+^/K^+^-ATPase activities, which is important for osmoregulation in fish subjected to salinity changes.^52^ Alternatively, elevated PL-DHA could be due to preprogrammed lipidomic remodeling in preparation for SW environments low in temperature and high in salinity.^51^

Our study has provided a broad overview of lipid composition and distribution in SW and FW salmon. By introducing a switch between FO and VO diets, we found that lipids changed much faster in the gut of SW salmon. This suggests that salmon have greater ability to absorb and process lipids after migration to the sea. On the other hand, FW salmon have a more active liver which synthesizes TGs in response to dietary shortage of LC-HUFA. The present study also suggests that SW salmon have a higher requirement for dietary DHA which needs to be incorporated into PLs for adaption to an SW environment.

The phenotypic, lipidomic, RNA-seq, and fatty acid data for each individual fish and tissue are publicly available on FAIRDOMHub (https://fairdomhub.org/investigations/79), an open source web platform for sharing scientific research assets, processes, and outcomes.^53^ Raw fastq files of RNA-Seq data are publicly available on the European Nucleotide Archive (ENA) under project accession number PRJEB24480. R and MATLAB codes for lipidomic and transcriptomic analysis were uploaded to Gitlab under project 3156778 (https://gitlab.com/digisal/GSF1_metabolomics).

## Supporting Information Available

Lipid class composition in the gut, liver, and muscle of SW and FW salmon during diet switch from FO to VO or vice versa; CPCA block score plots for lipid species of each lipid class between FW and SW salmon during diet switch; fatty acid composition in the gut, liver, and muscle of SW and FW salmon during diet switch from FO to VO or vice versa; composition of FO and VO diets for FW and SW salmon; and fatty acid profile of each diet (PDF)
