# Supplementary material for: Diet and Life Stage-Associated Lipidome Remodeling in Atlantic Salmon
Source: J Agric Food Chem. 2021 Mar 23;69(12):3787–96. doi: 10.1021/acs.jafc.0c07281 (PMC8041299; doi:10.1021/acs.jafc.0c07281)
Supplement: Supplementary file 1 — jf0c07281_si_001.pdf [file jf0c07281_si_001.pdf]

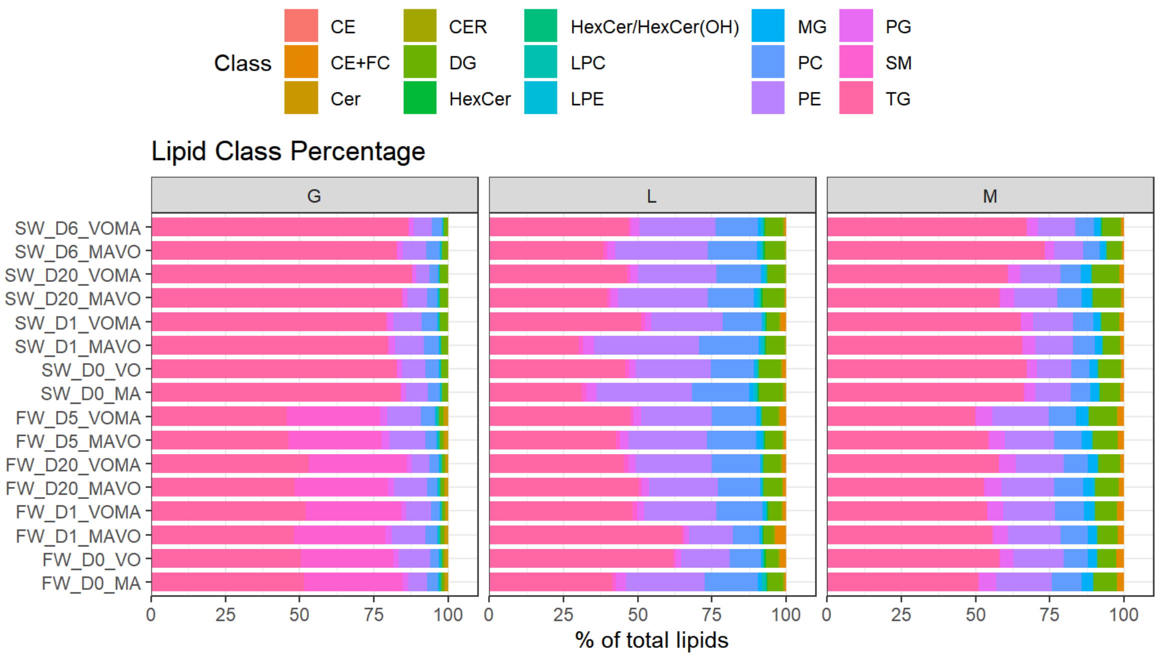

Supplementary Figure 1 Composition of lipid classes in gut (G), liver (L) and muscle (M) of seawater (SW) and freshwater (FW) salmon during diet switch from fish oil to vegetable oil (MAVO) or *vise versa* (VOMA). CE, cholesterol ester; FC, free cholesterol; Cer, Ceramides; HexCer, Hexosyl ceramide; DG, Diacylglycerol; LPC, Lysophosphatidylcholine; LPE, Lysophosphatidylethanolamine; MG, monoacylglycerol; PC, phosphatitylcholine; PE, phosphatidylethanolamine; PG, phosphatidylglycerol; SM, Sphingomyelin; TG, Triacylglycerol.

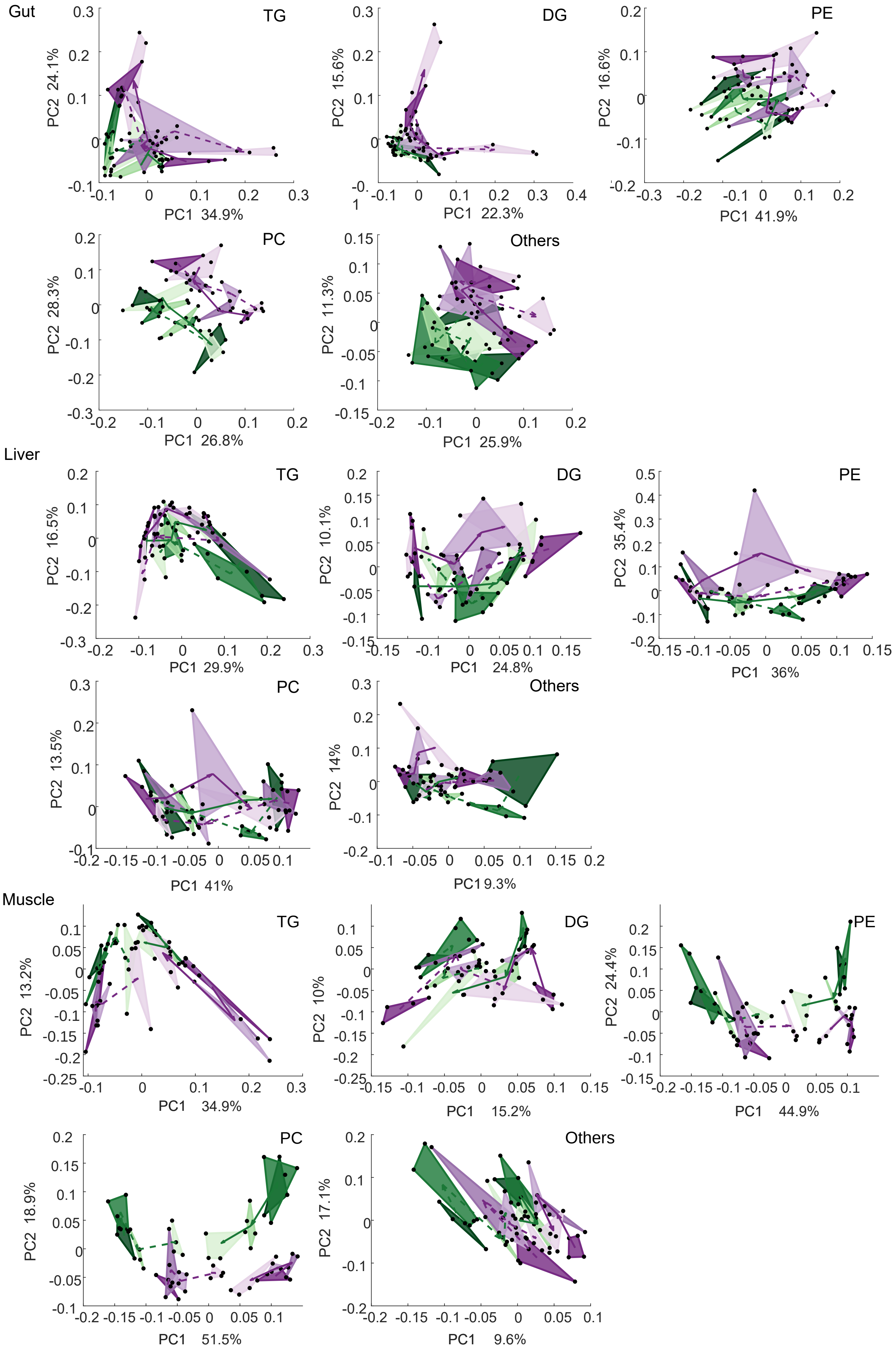

Supplementary Figure 2 PCA plots for lipid species of each lipid class between SW and FW salmon during diet switch.

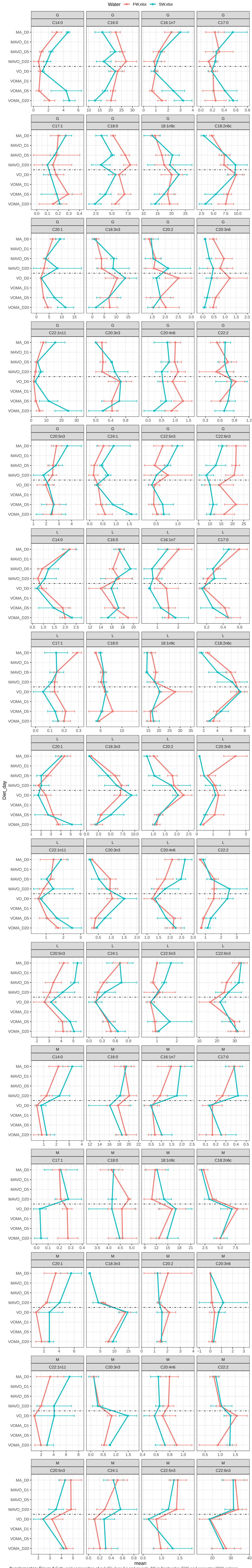

Supplementary Figure 3 Fatty acid composition of gut (G), liver (L) and muscle (M) in freshwater (FW) and seawater (SW) salmon during diet switch from fish oil to vegetable oil diet (MAVO) or *vice versa* (VOMA).

Supplementary Table 1 The composition of fish oil and vegetable oil diets (% of DW diet) for freshwater and seawater salmon and the fatty acid profile of each diet (% of total lipid fraction).

	Freshwater		Seawater	
	Fish Oil	Vegetable Oil	Fish Oil	Vegetable Oil
Feed composition % of total dry weight				
North Atlantic fishmeal	30.1	30.1	25.0	25.0
Plant meals	48.4	48.4	45.9	45.9
Additives	3.9	3.9	3.3	3.3
North Atlantic fish oil	17.6	1.2	25.8	1.8
Linseed oil	0.0	10.5	0.0	15.4
Palm oil	0.0	5.9	0.0	8.6
Total Protein	47	47	41	41
Total Lipid	22	22	31	31
Fatty acid composition % of total lipid fraction				
14: 0	6.4	1.5	6.5	1.6
15: 0	0.5	0.1	0.6	0.1
16: 0	15.4	18.5	15.3	19.5
18: 0	2.8	3.8	3.8	4.8
20: 0	0.1	0.2	0.1	0.1
Total Saturates	25.2	24.1	26.3	26.1
16:1n7	4.1	0.9	3.8	0.8
18:1n9	16.9	23.5	14.9	23.5
20:1n9	8	1.4	8.2	1.4
22:1n11	12	2.2	12.4	2.1
Total Monounsaturates	41	28	39.3	27.8
18:2n6	4.6	12.7	4.2	12.5
18:3n6	0.1	0.1	0.1	0.1
20:2n6	2.7	0.5	3	0.5
20:4n6	0.3	0.1	0.3	0.1
Total n-6 PUFA	7.7	13.4	7.6	13.2
18:3n3	2.11	28.8	2.5	26.8
20:3n3	0.2	0.1	0.3	0.1
20:5n3	5.8	1.3	6.2	1.4
22:5n3	0.9	0.2	0.8	0.2
22:6n3	10.7	2.3	11.7	2.7
Total n-3 PUFA	19.71	32.7	21.5	31.2
EPA+DHA	16.5	3.6	17.9	4.1

**The composition of diet and fatty acid were previously published in Gillard, et.al. 2018.[5]*
